# Organisational and management factors and related end-users’ perspectives relevant to newborn and stillbirth data at different levels of the health system: findings of the IMPULSE study in Uganda, Ethiopia, Tanzania, and the Central African Republic

**DOI:** 10.7189/jogh.15.04329

**Published:** 2025-12-05

**Authors:** Ilaria Mariani, Firehiwot Abathun, Ousman Mouhamadou, Jacqueline Minja, Rornald Muhumuza Kananura, Francesca Tognon, Mary Ayele, Giovanni Putoto, Tamrat Awell, Paolo Dalena, Sara Geremia, Lorenzo Giovanni Cora, Louise Tina Day, Donat Shamba, Peter Waiswa, Marzia Lazzerini

**Affiliations:** 1 Institute for Maternal and Child Health IRCCS Burlo Garofolo, WHO Collaborating Centre for Maternal and Child Health, Trieste, Italy; 2 Doctors with Africa CUAMM, Addis Ababa, Ethiopia; 3 Doctors with Africa CUAMM, Bangui, Central African Republic; 4 Ifakara Health Institute, Ifakara, Tanzania; 5 Demographic Dynamic and Population Health Unit, African Population and Health Research Center, Dakar, Senegal; 6 Doctors with Africa CUAMM, Padua, Italy; 7 Ministry of Health of Ethiopia: Strategic Affairs Executive Office; 8 Institute for Maternal and Child Health IRCCS Burlo Garofolo, Trieste, Italy; 9 University of Trieste, Trieste, Italy; 10 London School of Hygiene & Tropical Medicine, London, UK; 11 Makerere University of Public Health, Kampala, Uganda

## Abstract

**Background:**

As few studies systematically analysed organisational and management factors related to newborn and stillbirth data quality, we sought to identify specific gaps in these factors to provide evidence for planning tailored actions.

**Methods:**

We performed a cross-sectional survey in 12 regions and 4 city administrations in the Central African Republic (CAR), Ethiopia, Tanzania, and Uganda between November 2022 and July 2024, collecting data related to organisational and management factors at different health system levels through the Every Newborn – Measurement Improvement for Newborn & Stillbirth Indicators (EN-MINI) tools. We reported the results as frequencies/normalised PRISM scores, both on the overall sample and by country, and conducted exploratory subgroup analyses by region.

**Results:**

We included 151 sites (56 data offices; 95 facilities) and 108 health/data professional respondents. Availability of written documents describing the routine health information system (RHIS) mission, roles, and responsibilities (71.4% in the CAR to 94.1% in Tanzania; *P* = 0.380), and designated staff for internal data quality review (83.3% in Ethiopia to 100% in the CAR, Tanzania, or Uganda; *P* = 0.245) showed high percentages and low heterogeneity across countries at data office level. Most of the other measures explored – *i.e.* those related to governance, planning, financing, capacity development, relevant guidelines, data quality assurance systems, feedback mechanisms and supportive supervision – showed high heterogeneity across countries, with Ethiopia and Uganda, followed by Tanzania, showing the highest percentages, and the CAR showing the lowest. We observed low percentages in all countries at the data office level in the domains of financing (budget for RHIS supplies: 0% in the CAR to 35.3% in Tanzania; *P* = 0.079) and capacity development (availability of a report with RHIS training needs: 0% in the CAR to 41.2% in Tanzania; *P* = 0.333; training schedule: 17.6% in Tanzania to 42.9% in Uganda; *P* = 0.412). Subgroup analyses suggested high within-country heterogeneity. Needs for improvement in management and organisational factors were reported by most respondents (72.7% in Ethiopia to 100% in the CAR; *P* = 0.629).

**Conclusions:**

Our findings reveal a need for tailored interventions to improve organisational and management aspects at different levels of the health system, to ensure better quality and use of newborn and stillbirth data.

Every year, an estimated 2.3 million newborns die during the first 28 days of life, with around 1.9 million of these deaths being stillbirths, and nearly all (89%) occurring in low- and middle-income countries (LMICs) [1–3]. The sub-Saharan African region has the highest neonatal mortality and stillbirth rate in the world, with 27 neonatal deaths per 1000 live births [3,4] and an average stillbirth rate of 21 per 1000 total births [3]. Just a few years remains to meet the Sustainable Development Goal (SDG) 3.2 target for every country to reach a newborn mortality rate of 12 deaths or fewer per 1000 births [5]. While LMICs have made impressive progress towards this target, many African countries still have major constraints and are not on track for 2030 [6,7]. There is wide agreement among agencies (including the World Health Organization (WHO), United Nations Children’s Fund, United Nations Population Fund, United States Agency for International Development, and the World Bank) and researchers that gaps in high-priority data must be closed to end preventable stillbirth, deaths, and disabilities, ensure child health and well-being, and change how small and sick newborns are cared for [1,6,8–11].

The Performance of Routine Information System Management (PRISM) conceptual framework [12] and systematic reviews [13,14] identified organisational and management factors among the key inputs needed to achieve data quality and use in routine health information systems (RHISs). The former highlights that the effects of organisational and management factors on RHIS performance are both direct and indirect, as they also act through mediators, frequently through behavioural factors such as task competence and motivation [12,15].

According to the PRISM framework, organisational and management factors (Appendix S1 in the **Online Supplementary Document**) are relevant at different levels of health systems – *i.e.* at ministry of health (MoH), data office, and health facility levels – and include management of human and financial resources, logistics, planning, training, supervision, and leadership [12]. Different levels affect each other and contribute to the overall data quality and use. For example, MoH leadership affects the subnational data office, which in turn affect facilities and their data, while other management aspects at facility level, such as the lack of data quality assurance systems or the lack of feedback to staff, will affect data quality, and therefore impact response from the related data office, as well as at district and higher levels in a given health system [12,16].

Systematic reviews have shown that interventions to target organisational factors in LMICs are most often non-exhaustive and limited to training and supervision of data quality [17,18]. Research on organisational and management factors related to newborn and stillbirth data is very limited, with few investigations of PRISM organisational and management factors affecting newborn and stillbirth data quality in LMICs [19], despite studies [20,21] showing that big gaps for both newborn data availability and quality exist.

The IMProving qUaLity and uSE of newborn measures (IMPULSE) study aimed at filling some of these evidence gaps by assessing both newborn and stillbirth routine data quality and use and related inputs and process factors, defined according to the PRISM framework [12], at different levels of the health systems and across diverse regions in four African countries. It is a collaborative project led by following academic and research institutions: the London School of Hygiene & Tropical Medicine in the UK, the WHO Collaborating Center for Maternal and Child Health in Italy, the Ifakara Health Institute in Tanzania, the Makerere University of Public Health in Uganda, and the implementing agency Doctors with Africa *Collegio Universitario Aspiranti Medici Missionari* (*CUAMM*). The IMUPLSE study included an international advisory board, and a national advisory boards in each country.

This paper documents the findings of the IMPULSE study related to organisational and management factors relevant to newborn and stillbirth data in the CAR, Ethiopia, Tanzania, and Uganda, through direct observations of or data extraction from documents, and health and data professionals’ perspectives. Results related to newborn and stillbirth data quality and use and other process and input factors are reported in separate IMPULSE publications [22–25].

## METHODS

### Study design and participants

IMPULSE and this study were cross-sectional in design, so we reported our findings using the STROBE reporting guidelines for cross-sectional studies [26] (Appendix S2 in the **Online Supplementary Document**). It was conducted in a sample of health facilities and data sites in 12 regions and 4 city administrations in four countries: the CAR, Ethiopia, Tanzania and Uganda.

### Sampling methodology

We selected the country as the target geographical area for the lot quality assurance sampling survey methodology recommended by the Every Newborn – Measurement Improvement for Newborn & Stillbirth Indicators (EN-MINI) Performance of Routine Information System Management (PRISM) tools, which requires 19 or more health facilities to be assessed in each country [27]. We purposively selected 16 regions/city administrations across all countries based on a balance of the following three criteria:

1. heterogeneity, *i.e.* regions with different characteristics, including underperforming for maternal and neonatal mortality or humanitarian settings;

2. regions where the implementing agency, Doctors with Africa *CUAMM* had an office/project that could facilitate coordination or other easy-to-reach regions (well-connected or near to the capital city);

3. regions prioritised per request of the local MoH.

Among the 12 regions and 4 city administrations, ten (62.5%) were in difficult to reach/humanitarian setting (Appendices S3 and S4 in the **Online Supplementary Document**).

We only selected facilities providing a newborn inpatient care (*i.e.* comprehensive emergency obstetric and newborn care health facilities); however, as we could not do this in the CAR due to security reasons, we included seven facilities with lower levels of care. Our goal was to include facilities of different levels and types – including public, private, and not-for-profit institutions. We pre-defined a fixed number of facilities in each category for each region (Appendix S5 in the **Online Supplementary Document**), wherein we selected higher-level facilities among those with the higher number of deliveries. Health centres were, for practicality, selected in the same district as larger facilities. We also assessed one national hospital or the largest facility in each country. Lastly, we also included all data offices receiving data from the selected facilities at district, regional, and central level, identifying key data end-users in each site based on instructions included in the EN-MINI tools and approaching them for an interview. We pre-defined a minimum number of end-users of different types/levels in each facility according to its size.

We excluded sites in areas where active conflicts or where road conditions at the time of the assessment did not allow driving (Ethiopia: n = 5 health facilities and 1 subnational data office; Uganda: n = 1 health facility). Due to internal security constraints in the CAR, we focussed the assessment on Bangui city and the whole Health Region 7, and Health Regions 1 and 2.

### Data collection tools

We collected data between November 2022 and July 2024 using the open-access EN-MINI tools designed for government use, field tested in Tanzania and Ethiopia before their use. These include a tested adaptation of the PRISM tools, including ready-to-use digital data collection tools [12,28].

Here, we primarily used the Management Assessment EN-MINI Tool 4 for assessing the level of RHIS management functions, alongside additional questions from the RHIS Performance Diagnostic EN-MINI Tools 2a and 2b, which also capture organisational determinants such as indicator definitions and reporting guidelines, supervision and feedback mechanisms at data office (Tool 2a) and health facility (Tool 2b) levels [28]. Most data were directly observed and/or extracted from existing relevant documents (*e.g.* national guidelines, plans), except for key end-user perspective, which we collected with structured interviews with key staff based on tested tools, using mostly closed-ended questions regarding organisational and management factors at data office level (Tool 4) and supportive supervision at facility level (Tool 2b) [28].

Based on the EN-MINI tools standard operating procedures, key end-users were identified among data/health professionals involved in newborn data recording and reporting who were over the age of 18 years defined and belonged to the following subgroups:

− for data offices: individuals working in the health management information systems (HMIS) unit, the plan and policy unit, the quality unit, the maternal and child health programme staff, the regional health bureaus or the central MOH;

− in facilities: individuals in labour and delivery ward or neonatal intensive care unit coordinators (usually midwives or nurses), health management information system officers, plan and policy unit representatives.

Study coordinators created a list of all relevant professionals involved in newborn data recording, reporting, and use, at data office and facility level, which was used to identify one or two respondents for each site.

The EN-MINI tools were originally developed in English and were already available in Ki-Swahili for Tanzania. The IMPULSE study further optimised, field tested, and translated them into Amharic (for use in Ethiopia) and French (for use in the CAR). This was done with the help of local translators in each country, with revisions by study coordinators. A team of trained data collectors piloted the tools under the supervision of a study coordinator in each country. Any remaining doubt on data collection was addressed in real time during data collection among the study team (*via *WhatsApp communication or in-person discussions with the study coordinator).

### Data management and data quality assurance

Two authors (LTD and JM) trained the study coordinators (FA, MA, MKR, OM) and other IMPULSE team members (FT and ML) in the English version of the EN-MINI tools. The study coordinators trained teams of 3–6 data collectors in each country, operationalising them with field practices; meetings to discuss and clarify doubts and questions; a file where all questions and answers were recorded; and a WhatsApp group to solve any remaining issue in real-time.

Standard operating procedures for data collection were predefined in dialogues with study coordinators. Data were collected in respondents’ and data collectors’ language of choice (Amharic, English, French, Swahili). Key end-users received an IMPULSE information sheet and gave written consent prior to participation. Using password-protected tablets or phones, data were directly entered into an Open Data Kit forms which included built-in validations to check for data completeness and plausibility, after which they were uploaded to a secure encrypted server hosted by SurveyCTO (Dobility, Inc., Washington, D.C., USA) through a community license [29]. No individual identifiers other than the region and site name were collected.

Study coordinators (FA, JM, MA, MKR, OM), supervised by FT, monitored and evaluated data collection processes daily using a customised, field tested Excel sheet, ensuring timeliness, completeness, and arrival at an adequate sample size. Missing data or implausible data were discussed in real-time. Data were downloaded in each country, checked to ensure no key informant identifiers, and securely transferred to the study group.

The IMPULSE study data analysts (IM, PD, SG) performed four rounds of interim analyses during data collection to assess data completeness, internal consistency, and plausibility, discussing their results after each round and optimising data collection (*e.g.* if there were missing data) accordingly.

Final results were then cross-checked at the data analysis phase against those of the of the EN-MINI-PRISM analysis tool, which is included in the EN-MINI tools resources [28]. Additionally, a data validation national workshop was held in each country to discuss IMPULSE study phase 1 findings and to identify country-specific priorities for actions and related activities with local stakeholders, such as MoH and national scientific associations.

### Data analysis

We performed our analyses based on the published PRISM User’s Kit, which has been used in >40 countries over >10 years [27]. We first performed descriptive analyses, determining the frequency distribution for each organisational and management measures for the six domains: governance, planning, finance, capacity development, quality (including guidelines, data quality assurance systems, and feedback mechanisms), and supervision. These were compared through a χ^2^ test or Fisher’s exact test, as appropriate. We calculated average scores for data quality control and quality supervision per the PRISM User’s Kit [27] (Appendix S6 in the **Online Supplementary Document**). From end-users’ interviews, we analysed only the closed-ended question related to the perceived need for improvement in organisational and management factors, and will report on the remaining findings elsewhere. Lastly, we performed exploratory stratified analyses by country and region.

A two-tailed *P*-value <0.5 indicated statistical significance. We performed all analyses in *R*, version 4.1.1 (R Foundation for Statistical Computing, Vienna, Austria).

### Ethical aspects

Data collection was conducted according to General Data Protection Regulation regulations. Anonymity was ensured by not collecting any information that could disclose participants’ identity. Data were transmitted and stored in password protected tablets, and uploaded into encrypted servers. Paper documents were stored in locked filing cabinets. For interviews, staff were informed of the study objectives and methods, including their rights in declining participation, prior to enrolling *via* an information sheet, with each providing written consent expressing their voluntary willingness to participate the survey.

## RESULTS

### Sample characteristics

We collected data from 151 sites, or more specifically, 56 data offices (using EN-MINI Tools 4 and 2a) and 95 facilities (using EN-MINI Tool 2b). The sample size varied by measure based on what tool was used for data collection, with most of the measures reported here being collected with the EN-MINI Tool 4, focussing on data offices (Appendix S7 in the **Online Supplementary Document**).

We included both central/regional (n = 11, 19.6%) and district data offices (n = 45, 80.4%), as well as 17 (17.9%) third-level, 39 (41.1%) second-level, and 39 (41.1%) first-level referral facilities. A total of 108 end-users were interviewed – 56 from data offices and 52 from facilities.

### Governance, planning, financing, capacity development

**Figure 1:**
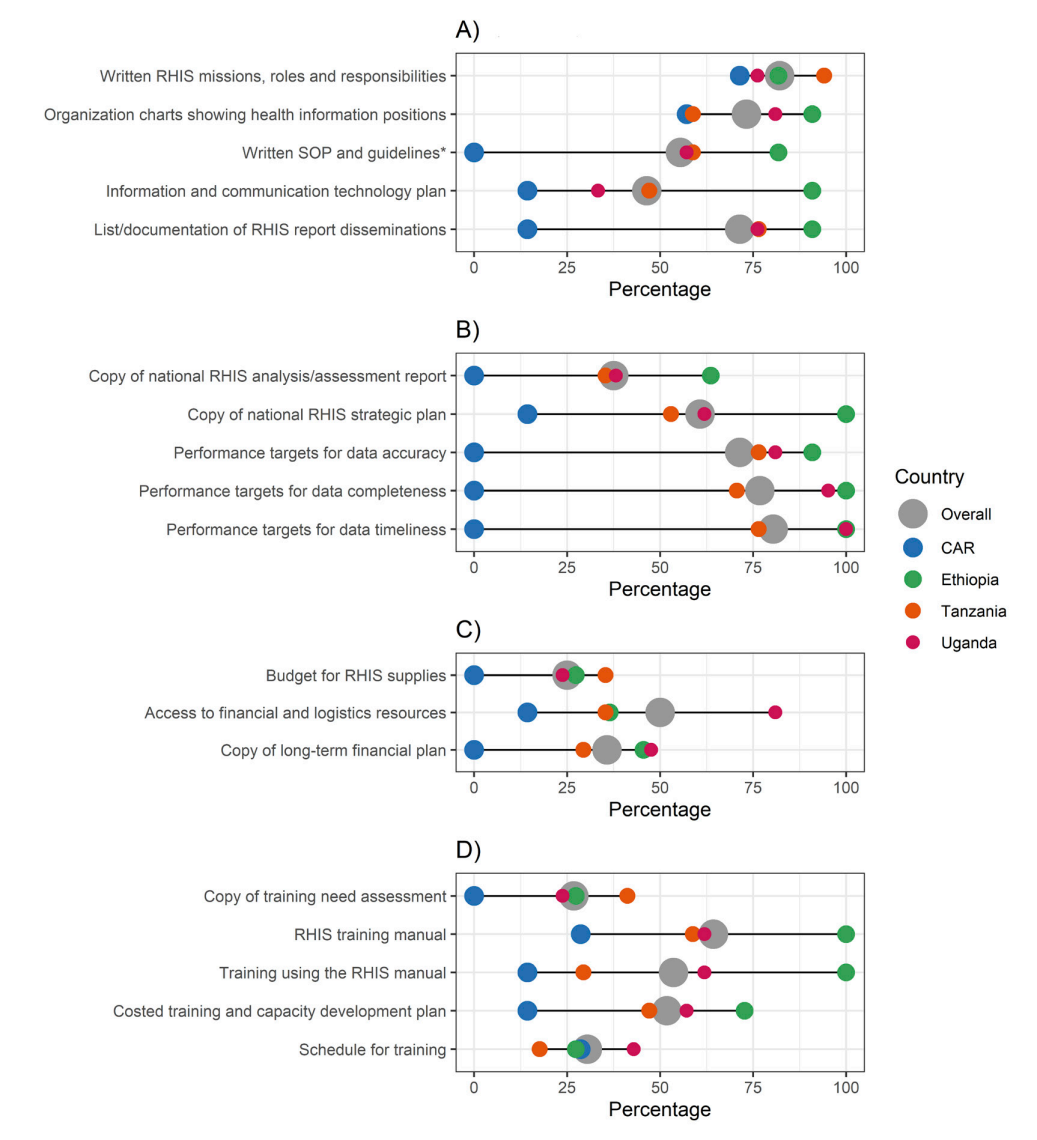
IMPULSE study: governance, planning, financing, capacity development at data office level. Each dot represents the value for one country; differences in dot size are due to the need to graphically represent overlapping percentages. **Panel A.** Governance. **Panel B.** Planning. **Panel C.** Financing. **Panel D.** Capacity development. *Written SOP and procedural guidelines for RHIS that included: data definitions including newborn and stillbirth data elements/measures; data collection and reporting including newborn and stillbirth data elements/measures; data aggregation, processing, and transmission including newborn and stillbirth data elements/measures; data analysis, dissemination, and use including newborn and stillbirth data elements/measures; data quality assurance including newborn and stillbirth data elements/measures; master facility list; International Classification of Diseases codes relevant to newborns and stillbirths; data security; data storage; and performance improvement processes. CAR – Central African Republic, RHIS – routine health information system, SOP – standard operating procedures.

**Figure 2:**
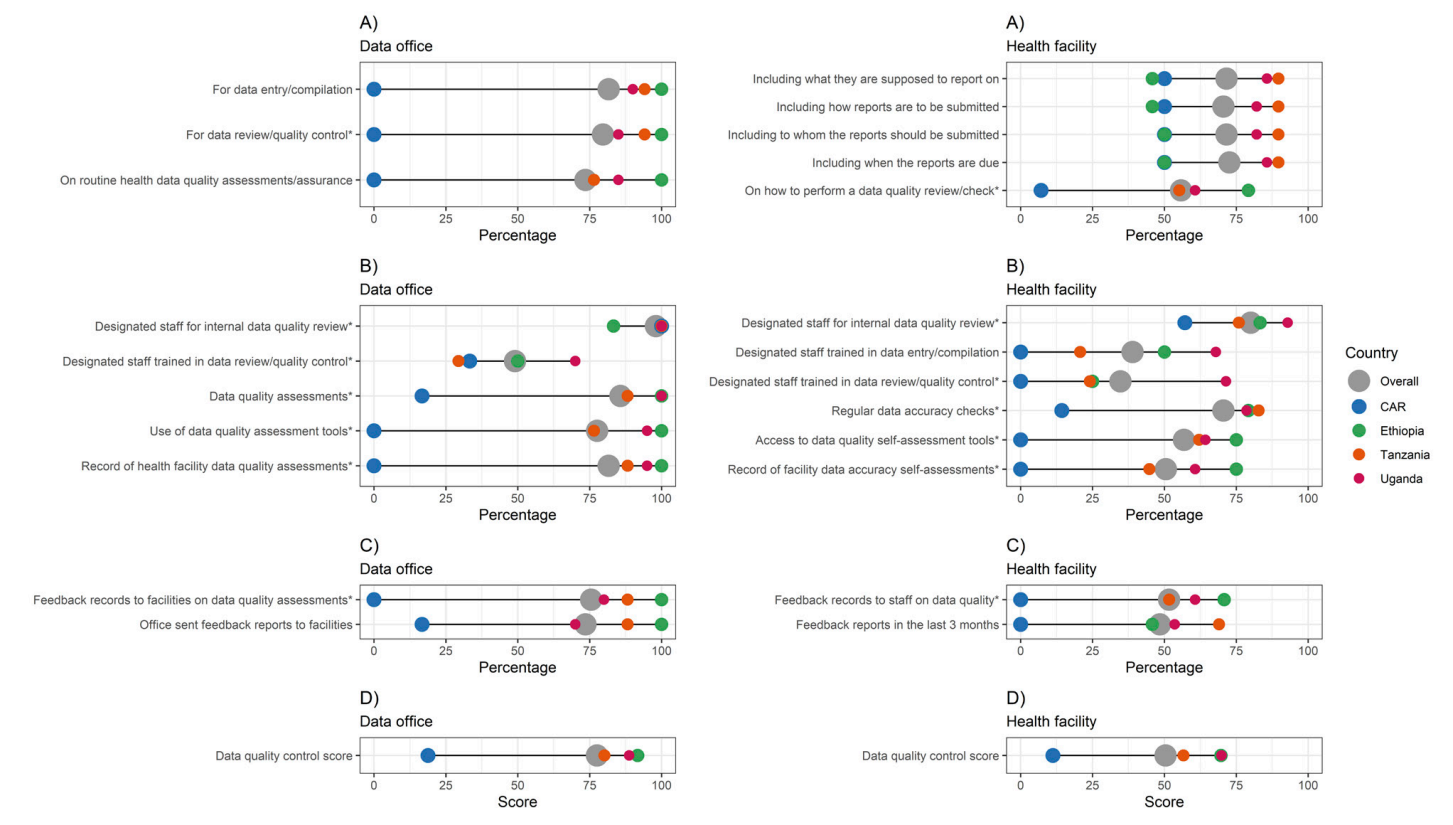
IMPULSE study: guidelines, data quality assurance systems and feedback mechanisms. Each dot represents the value for one country; differences in dot size are due to the need to graphically represent overlapping percentages. **Panel A.** Guidelines. **Panel B.** Data quality assurance system. **Panel C.** Feedback mechanism. **Panel D.** PRISM score on data quality control. *Variables contributing to the data quality score, calculated according to the PRISM user's kit [27]. CAR – Central African Republic, PRISM – Performance of Routine Information System Management.

**Figure 3:**
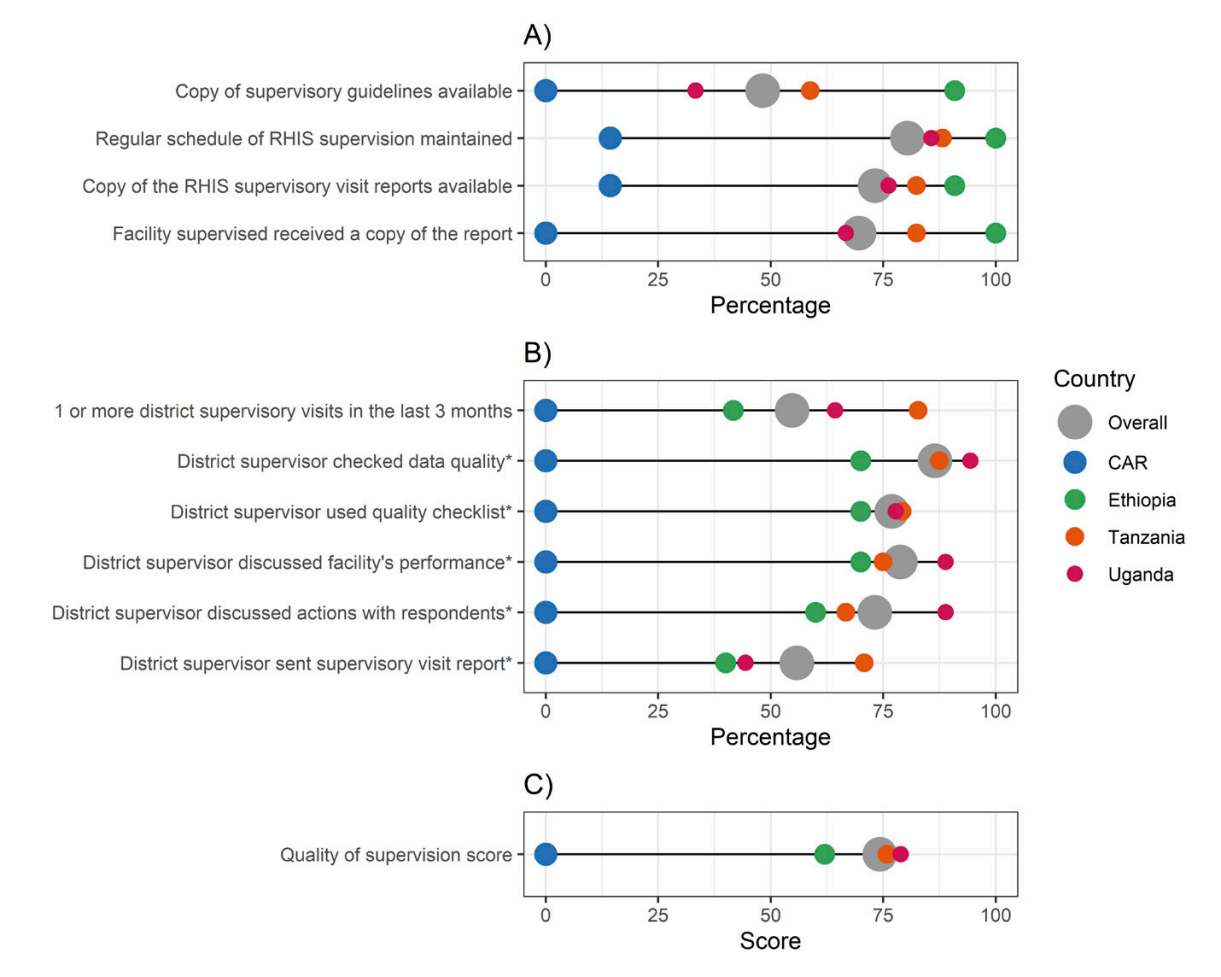
IMPULSE study: supportive supervision from data offices. Supportive supervision at data office (**Panel A**) and health facility (**Panels B and C**) level. Each dot represents the value for one country; differences in dot size are due to the need to graphically represent overlapping percentages. **Panel A.** Supportive supervision: key aspects. **Panel B.** Supportive supervision: quality. **Panel C.** Supportive supervision: PRISM score. *Measures contributing to the PRISM score on quality of supervision, calculated according to the PRISM user's kit [27]. CAR – Central African Republic, PRISM – Performance of Routine Information System Management, RHIS – routine health information system

We observed high heterogeneity across countries in explored measures (**Figure 1**, **Figure 2**, **Figure 3**; Appendices S8–10 in the **Online Supplementary Document**), with Ethiopia (**Figure 1**, **Figure 2**, **Figure 3**) generally performing better than other countries, followed by Uganda and Tanzania, and the CAR showing lower frequencies on most measures, with few exceptions.

Four measures had similar percentages across countries. The presence of a written document describing the RHIS mission, roles, and responsibilities had relatively high percentages in all countries, ranging from 71.4% in the CAR to 94.1% in Tanzania (*P* = 0.380). Conversely, all countries had relatively low percentages in: the availability of a budget for RHIS supplies (0% in the CAR to 35.3% in Tanzania; *P* = 0.079); the availability of a copy of RHIS training needs assessment (from 0% in the CAR to 41.2% in Tanzania; *P* = 0.333); the availability of a schedule for planned training (from 17.6% in Tanzania to 42.9% in Uganda; *P* = 0.412).

All other measures showed high between-country variability. In the domain of governance (**Figure 1**, Panel A), the availability of a comprehensive list of standard operating procedures and guidelines varied highly (from 0% in the CAR to 81.8% in Ethiopia; *P* = 0.006). In the 15 data offices with a partial presence of standard operating procedures and written guidelines, areas not covered by guidelines were mainly related to data security (60.0% data offices; *P* = 0.385); data analysis, dissemination, and use (46.7% data offices; *P* = 0.017); data aggregation, processing, and transmission (40.0% data offices; *P* = 0.025); or to master facility list (46.7% data offices; *P* = 0.271); or International Classification of Diseases codes (46.7% data offices; *P* = 0.271) (Appendix S8 in the **Online Supplementary Document**).

The domain of planning showed high heterogeneity across countries in all the assessed measures (**Figure 1**, Panel B). The financing domain had frequencies below 50% in all countries except for the access to financial and logistics resources for RHIS supervision in Uganda (81.0%) (**Figure 1**, Panel C). Most measures in the domain of capacity development (*i.e.* RHIS training manual, training using the RHIS manual, training and capacity development plan) varied highly across countries (**Figure 1**, Panel D**)**.

### Guidelines, data quality assurance systems, and feedback mechanisms

Few measures had low variability among measures related to guidelines, data quality assurance systems, and feedback mechanisms on data quality assessments both at data office and health facility levels **(****Figure 2**, Panels A–C; Appendix S9 in the **Online Supplementary Document**). In the domain of data quality assurance systems, we observed low heterogeneity for designated staff for internal data quality review at the data office (from 83.3% in Ethiopia to 100% in the CAR, Tanzania and Uganda; *P* = 0.245). The availability of designated staff for internal data quality review and designated staff trained in data review and quality control were collected both at data office and health facility levels, with lower overall percentages (80.0% and 34.7%, respectively) and higher heterogeneity across countries at facility level compared to data offices (overall frequencies of 98% and 49%. respectively).

The PRISM average summary score on data quality control (**Figure 2**, Panel D) showed a large gap in between the CAR (18.8%) and the other three countries both at the data office level (80.2% in Tanzania, 88.8% in Uganda, 91.7% in Ethiopia) and at the health facility level (11.2% in the CAR, 56.7% in Tanzania, 69.6% in Ethiopia, 69.9% in Uganda).

### Supportive supervision

Supportive supervision measures at data office level varied widely, with the CAR showing the lowest percentages (lower than 15% on all measures) and Ethiopia the highest (more than 90% for all measures). Copies of RHIS supervisory guidelines and checklists were scarcely present in the CAR (0%), Uganda (33.3%), and Tanzania (58.8%) (**Figure 3**, Panel A; Appendix S10 in the **Online Supplementary Document**).

Supervisory visits were conducted at least once in the previous three months in Tanzania, Uganda, and Ethiopia, in 82.8%, 64.3% and 41.7% of health facilities, respectively (**Figure 3**, Panel B). Among them, measures of supervision quality showed low variability and frequencies ≥70%, except for the report/written feedback sent by supervisors, with frequencies below 50% in Ethiopia (40.0%) and in Uganda (44.4%). No supervisory visit was conducted in the CAR.

The PRISM average score on quality of supervision at health facility level (**Figure 3**, Panel C) showed relatively high frequencies above 75.0% in Uganda (78.9%) and Tanzania (75.8%), and a score of 62.0% in Ethiopia and 0% in the CAR.

### End-users’ perspectives

Among 108 end-users interviewed, 100% of data office staff interviewed in the CAR, 82.4% in Tanzania, 81.0% in Uganda, 72.7% in Ethiopia (*P* = 0.629) indicated a need for improvement in the organisational and management factors. About one out of three staff in the CAR (28.6%) and Ethiopia (36.4%) reported the need for a major improvement in the RHIS. Specific to supportive supervision at the health facility level, a need for improvement was requested by 66.7% of end-users in Tanzania, 60.0% in Ethiopia, and 44.4% in Uganda, in facilities where supervisory visits occurred (Appendix S11 in the **Online Supplementary Document**).

### Subgroup exploratory analyses by region

We also identified large heterogeneity within countries when analysing data by region (Appendices S8–11 in the **Online Supplementary Document**). Results from city administrations showed higher percentages than in the regions on most measures, with some exceptions in the measures of supervisions for all countries, among a few others. Specifically, in the CAR, guidelines-related measures at health facility level had higher percentages in Health Region 1 compared to Bangui City Administration, while in Tanzania, some measures of the data quality assurance system and feedback at health facility level were higher in all regions compared to the Dar es Salaam City Administration.

## DISCUSSION

This is the first multi-country study exploring, per the PRISM framework, a comprehensive list of organisational and management factors relevant to newborn and stillbirth data at different health system levels (**Box 1**). Our findings suggest that organisational and management factors vary highly across the included countries, both at data office and health facility levels. We observed measures showing good organisation and management in all countries, with Ethiopia showing the highest frequencies in most of the analysed measures, followed by either Uganda or Tanzania. We simultaneously noted several organisational and management gaps for all countries, with the CAR showing many gaps, and with most health data office staff in each country reporting a need for improvement.

Box 1 **Key study findings**

What was known before this study?

– High quality newborn and stillbirth healthcare data are needed for action to end preventable mortality and morbidity and reach globally agreed goals by 2030.

– Organisational and management factors are key determinants of newborn and stillbirth data quality and use in the RHIS and need to be tracked and monitored.

– Little is known regarding RHIS-related organisational and management factors in the CAR, Ethiopia, Tanzania, and Uganda.

What did we find and what does it mean?

– Organisational and management factors varied highly across countries, both at data office and health facility levels.

– Common strengths in all countries were the availability of written documents describing the RHIS mission, roles, and responsibilities, and the availability of designated staff for internal data quality review.

– Common gaps were related to the domains of financing and capacity development.

– Most end-users requested improvements in organisational and management factors in all countries.

– Other IMPULSE publications are reporting on staff performance in using eRHIS and on human resources.

What novelties does this study bring?

– First four-country multi-regional study in Africa to explore the RHIS organisational and management factors for high-quality newborn and stillbirth indicator data in high-mortality settings, utilising a standardised methodology and many variables.

What is next for implementation?

– Optimise organisational and management factors to streamline newborn and stillbirth indicator data both in health facilities and data offices, considering the context-specific needs.

What research gaps remain?

– Implementation research to identify sustainable interventions for improving RHIS organisational and management factors in high mortality settings.

– The PRISM methodology could be further optimised, particularly by identifying aggregate indicators that can be used in multivariate analyses to identify factors related to best/worst performance.

Some of these gaps have been acknowledged already in local annual plans/guidelines from Ethiopia, Tanzania, and Uganda [30–32] and partially in other reports related to the CAR [33], with several efforts undertaken recently to address them. The good results observed here on measures related to the availability of written documents describing the RHIS mission, roles, and responsibilities, as well as the availability of designated staff for internal data quality review at data office levels in Ethiopia, Tanzania, and Uganda, are in line with efforts of the MoH of Ethiopia to strengthen health information system governance, establish standards and guidelines for digital health solutions, data access, storage, processing, information, exchange, and sharing, and to maximise the utilisation of guidelines [31]. Similarly, health information system guidelines were published Tanzania in 2019 as ‘guiding reference for managing the flow of health data through an integrated HIS’ [30]. In Uganda, existing guidelines aim at ensuring data quality across all levels of the health system [32].

Despite such good systems being in place, there remain gaps in data quality and use in the three countries [22,25]. Our findings suggest that implementation of such guidelines was still incomplete, as shown by the low observed frequencies in financing and capacity development domains, as well as in specific indicators from governance, planning, data quality assurance systems, and feedback mechanisms, and supervision domains. Misalignment between guidelines/policies and real practice, limited resources, ineffective monitoring, human resources shortage, and cultural resistance might be just some of the bottleneck factors inhibiting guideline implementation [34–36]. Further research into these factors within the included countries is urgently needed, as it could help with the development of priority actions. Lastly, no existing guidelines on data quality have been published by the MoH in the CAR.

Previous studies reported similar findings to ours, although they most often focussed on measures not specific to newborns/stillbirth. For example, in a study involving 386 department heads in 83 health facilities in Northwest Ethiopia, written feedback on data quality provided by data offices was lacking in 66.6% of cases, while in alignment with our findings, RHIS supervision visits were reported by 41% of the department heads, of which almost half provided written reports on weaknesses and strengths of RHIS performance [37]. Rates of supervision reports or written feedback below 30% in Ethiopia were noted in two other studies conducted in Ethiopia [13,38]. Another study from Tanzania highlighted limited planning skills of district planning teams and inadequate knowledge on planning guidelines as key gaps that need to be addressed [39]. Existing literature, however, scarcely assessed needs for improvement in the organisational and management factors among end-users working in newborn area. However, other research assessing RHIS strengthening areas found similar results; for example, 60% of 111 respondents from health facilities in Cameroon requested RHIS strengthening or development for management and governance areas [40].

There may be many contextual factors contributing to gaps in organisation and management. Related to the planning and financing domains, lack of sufficient funding was commonly reported in other studies in African countries [31,39,41]. Difficulties related to the long-term RHIS financial planning may be connected to the multiple competing priorities, the need to pool different types of inconstant financing from donors, and corruption [31,39,42,43]. Multiple other factors should be acknowledged among the underlying determinants of the observed gaps, including socioeconomic, political, and historical aspects [13,40,41,44]. We particularly note the political instability, the reduced health expenditure *per capita*, the low number of medical doctors, and the ongoing conflict and related emergencies in the CAR, which may also contribute to the lack of improvement in the stillbirth and neonatal mortality rates there compared to other countries [43,45]. Recent conflicts may also have affected measures in included conflict regions in Ethiopia (Appendix S4 in the **Online Supplementary Document**) [46].

In line with previous research [47–49], exploratory analyses showed regional differences within countries, even in the CAR, indicating the presence of some well-performing areas in the analysed countries and suggesting that improvements are therefore feasible and achievable. Other input factors affecting data quality and use and comprehensively reporting country data are more widely described in other IMPULSE publications [22–25].

The findings our study inform researchers, end-users, and stakeholders on the strengths and gaps in organisational and management factors related to newborn and stillbirth data and calls for targeted interventions. All domains and PRISM measures explored in this study may be important in principle, as highlighted by a large body of literature [13,50–52], although specific action require prioritisation. National workshops were held in each country in the IMPULSE study to identify local priorities, in dialogue with local stakeholders such as MoHs and national scientific associations. Interventions discussed in these workshops varied in complexity and resources required for implementation. For example, one relatively low-resource and non-time-consuming action suggested in the CAR may be the publication of national guidelines on newborn data quality, including on relevant aspects on governance. In all countries, a better system of feedback and supervision was recognised as important for improving quality of routine newborn and stillbirth data, which has also been underlined by other studies [16,53]. 

Other interventions discussed with stakeholders included the availability of designated staff trained in data entry and data quality control. However, this would require important resources. Broader, more complex and long-term interventions with a positive expected impact on the RHIS may be needed, including increased domestic investment in maternal and newborn health, political stability, efforts for conflict resolution and peaceful coexistence with different ethnic groups. Financial support from donors seems to still be critical, particularly for complex interventions and in countries such as the CAR. The feasibility of these interventions may be increased by coordinating interventions with other existing initiatives (such as the WHO’s Data for Health initiative), although recent funding cuts have put at risk the existence of many previously well-established programmes [54,55]. In parallel, more implementation research is needed to identify culturally appropriated and sustainable interventions to improve organisational and management factors at different levels of the health system related to newborn and stillbirth data in the RHIS. Our findings and those of other IMPULSE reports could thus help researchers identify key areas that require improvements and develop context-specific quality improvement initiatives.

Our results are not directly generalisable to other settings; however, our assessment methods could easily be replicated in other sites. We acknowledged the limited sample size in the CAR, driven by security reasons, as another limitation of this study. This reduced generalisability of data, and together with the inclusion of seven basic emergency obstetric and newborn care facilities, may have contributed to the observation of a low quality of organisational and management factors in the CAR. Including *CUAMM*-supported regions may have limited representativeness, as *CUAMM*-supported facilities are located in humanitarian settings or regions with chronic constraints, but are usually better resourced than many other facilities within these countries. Gaps in organisational and management factors in such facilities, therefore, suggests that the broader national situation may be even more concerning. While we recommend extending the assessment to additional facilities, it is reasonable to expect that similar or greater challenges would be observed. Further research in the four countries is needed to fully explore organisational and management factors in the regions not included in our analysis. Another limitation of our research is the lack of a summary dependent variable recognised by PRISM framework [12] and the PRISM manual of analysis [27] that discouraged us from utilising regression models. Also, EN-MINI Tools 2 and 4 did not collect sociodemographic information or data related to role or cadre from end-users [28]; however, based on the EN-MINI instructions, we interviewed key data end-users in each site.

Despite these limitations, we note that our study covered included diverse geographical areas, and different levels and types of facilities/data offices. We also conducted our assessment per the PRISM framework and methodology [12,27], which had been previously applied in many countries and adapted for the assessment of stillbirth and newborn data quality [28,56]. We also assessed a large number of variables and collected most data through direct observation.

## CONCLUSIONS

While noting several strengths, we also observed several organisational and management gaps related to newborn and stillbirth data in the RHIS at all levels in all study countries, and in the CAR, in particular. A need for improving organisational and management aspects related to the RHIS, as well as supportive supervision, were also highly reported by end-users in all countries. Our findings may serve countries and drive locally-led quality improvement initiatives and context-specific tailored actions for each organisational and management domain. More broadly, alongside constant monitoring, organisational and management aspects require more focussed attention and intervention, in order to achieve a better quality and use of RHIS data, useful for reducing newborn mortality and stillbirth rates.

## Additional material


Online Supplementary Document
