# Supplementary material for: Organisational and management factors and related end-users’ perspectives relevant to newborn and stillbirth data at different levels of the health system: findings of the IMPULSE study in Uganda, Ethiopia, Tanzania, and the Central African Republic
Source: J Glob Health. 2025 Dec 5;15:04329. doi: 10.7189/jogh.15.04329 (PMC12677244; doi:10.7189/jogh.15.04329)
Supplement: Online Supplementary Document [file jogh-15-04329-s001.pdf]

Supplement to: Mariani I, Abathun F, Mouhamadou O, Minja J, Kananura RM, Tognon F, Ayele M, Putoto G, Awell T, Dalena P, Geremia S, Cora LG, Day LT, Shamba D, Waiswa P, Lazzerini M. Organisational and management factors and related end-users' perspectives relevant to newborn and stillbirth data at different levels of the health system: findings of the IMPULSE study in Uganda, Ethiopia, Tanzania, and the Central African Republic. J Glob Health. 2025;15:04329.

Table of contents

Appendix S1. PRISM conceptual framework..2

Appendix S2. The Strengthening the Reporting of Observational Studies (STROBE) Checklist.....3

Appendix S3. Geographical distribution of the regions included in the IMPULSE study..............5

Appendix S4. Characteritics of the regions..6

Appendix S5. Sampling criteria..7

Appendix S6. Formulas for the composite indicators according to the the PRISM User's Kit 2019 8

Appendix S7. Sample characteristics by IMPULSE administrative unit and regions.....................9

Appendix S8. Governance, planning, financing, capacity development at data office level * ...11

Appendix S9. Guidelines, data quality assurance systems and feedback mechanisms 13

Appendix S10. Supportive supervision from data office..15

Appendix S11. End-users' perspectives...16

Appendix S1. PRISM conceptual framework

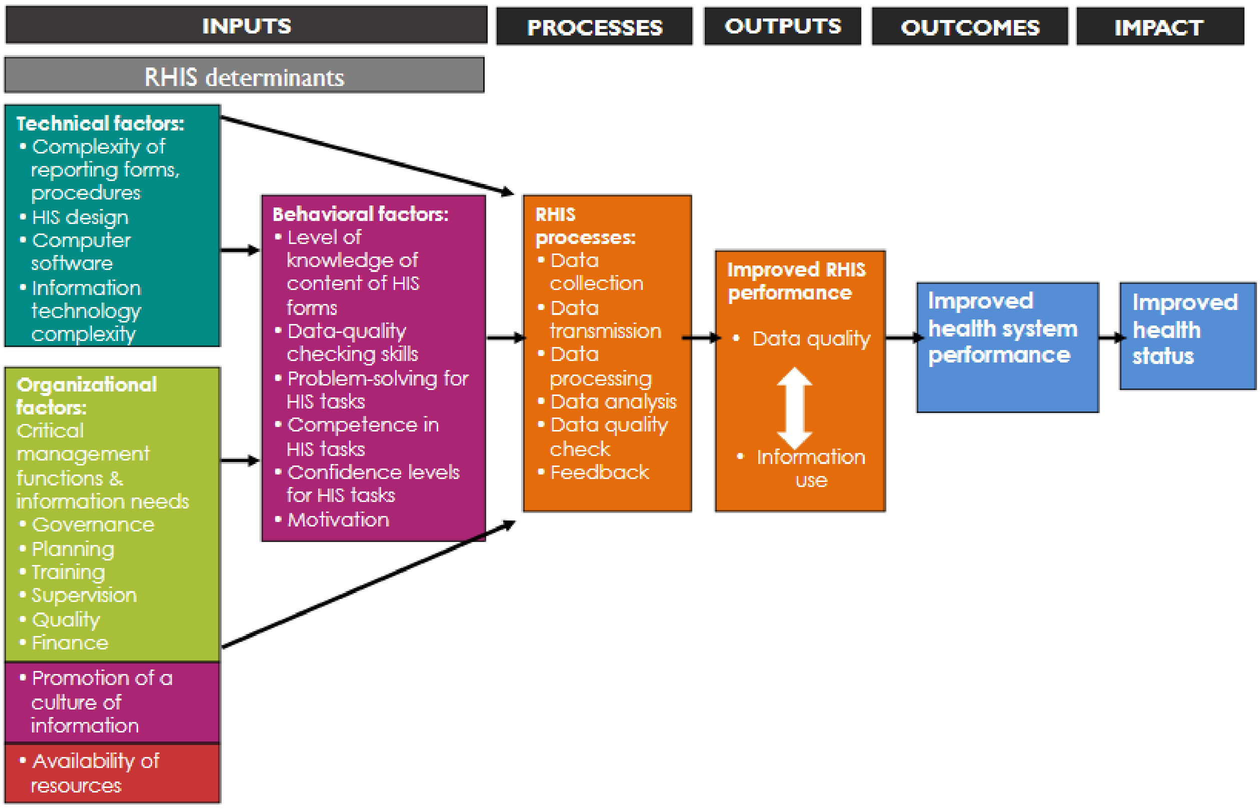

Reference: Aqil A, Lippeveld T, Hozumi D. PRISM framework: a paradigm shift for designing, strengthening and evaluating routine health information systems. Health Policy Plan. 2009;24:217–28. doi:10.1093/heapol/czp010

Abbreviations: PRISM = Performance of Routine Information System Management

Appendix S2. The Strengthening the Reporting of Observational Studies (STROBE) Checklist

	Item No	Recommendation	Pages
**Title and abstract**	1	(*a*) Indicate the study's design with a commonly used term in the title or the abstract	4
		(*b*) Provide in the abstract an informative and balanced summary of what was done and what was found	4
**Introduction**			
Background/rationale	2	Explain the scientific background and rationale for the investigation being reported	5-6
Objectives	3	State specific objectives, including any prespecified hypotheses	6
**Methods**			
Study design	4	Present key elements of study design early in the paper	6
Setting	5	Describe the setting, locations, and relevant dates, including periods of recruitment, exposure, follow-up, and data collection	6-8
Participants	6	(*a*) Give the eligibility criteria, and the sources and methods of selection of participants	6-7
Variables	7	Clearly define all outcomes, exposures, predictors, potential confounders, and effect modifiers. Give diagnostic criteria, if applicable	8
Data sources/ measurement	8*	For each variable of interest, give sources of data and details of methods of assessment (measurement). Describe comparability of assessment methods if there is more than one group	8
Bias	9	Describe any efforts to address potential sources of bias	7-8
Study size	10	Explain how the study size was arrived at	6-7
Quantitative variables	11	Explain how quantitative variables were handled in the analyses. If applicable, describe which groupings were chosen and why	8-9
Statistical methods	12	(*a*) Describe all statistical methods, including those used to control for confounding	8-9
		(*b*) Describe any methods used to examine subgroups and interactions	8-9
		(*c*) Explain how missing data were addressed	8-9
		(*d*) If applicable, describe analytical methods taking account of sampling strategy	NA
		(*e*) Describe any sensitivity analyses	-
**Results**			

Participants	13*	(a) Report numbers of individuals at each stage of study—eg numbers potentially eligible, examined for eligibility, confirmed eligible, included in the study, completing follow-up, and analysed	9-10
		(b) Give reasons for non-participation at each stage	-
		(c) Consider use of a flow diagram	-
Descriptive data	14*	(a) Give characteristics of study participants (eg demographic, clinical, social) and information on exposures and potential confounders	9-10
		(b) Indicate number of participants with missing data for each variable of interest	Appendix
Outcome data	15*	Report numbers of outcome events or summary measures	10-13
Main results	16	(*a*) Give unadjusted estimates and, if applicable, confounder-adjusted estimates and their precision (eg, 95% confidence interval). Make clear which confounders were adjusted for and why they were included	10-12
		(*b*) Report category boundaries when continuous variables were categorized	-
		(*c*) If relevant, consider translating estimates of relative risk into absolute risk for a meaningful time period	-
Other analyses	17	Report other analyses done—eg analyses of subgroups and interactions, and sensitivity analyses	13
**Discussion**			
Key results	18	Summarise key results with reference to study objectives	13
Limitations	19	Discuss limitations of the study, taking into account sources of potential bias or imprecision. Discuss both direction and magnitude of any potential bias	15
Interpretation	20	Give a cautious overall interpretation of results considering objectives, limitations, multiplicity of analyses, results from similar studies, and other relevant evidence	13-15
Generalisability	21	Discuss the generalisability (external validity) of the study results	13-15
**Other information**			
Funding	22	Give the source of funding and the role of the funders for the present study and, if applicable, for the original study on which the present article is based	16

Notes: * Give information separately for exposed and unexposed groups.

Appendix S3. Geographical distribution of the regions included in the IMPULSE study

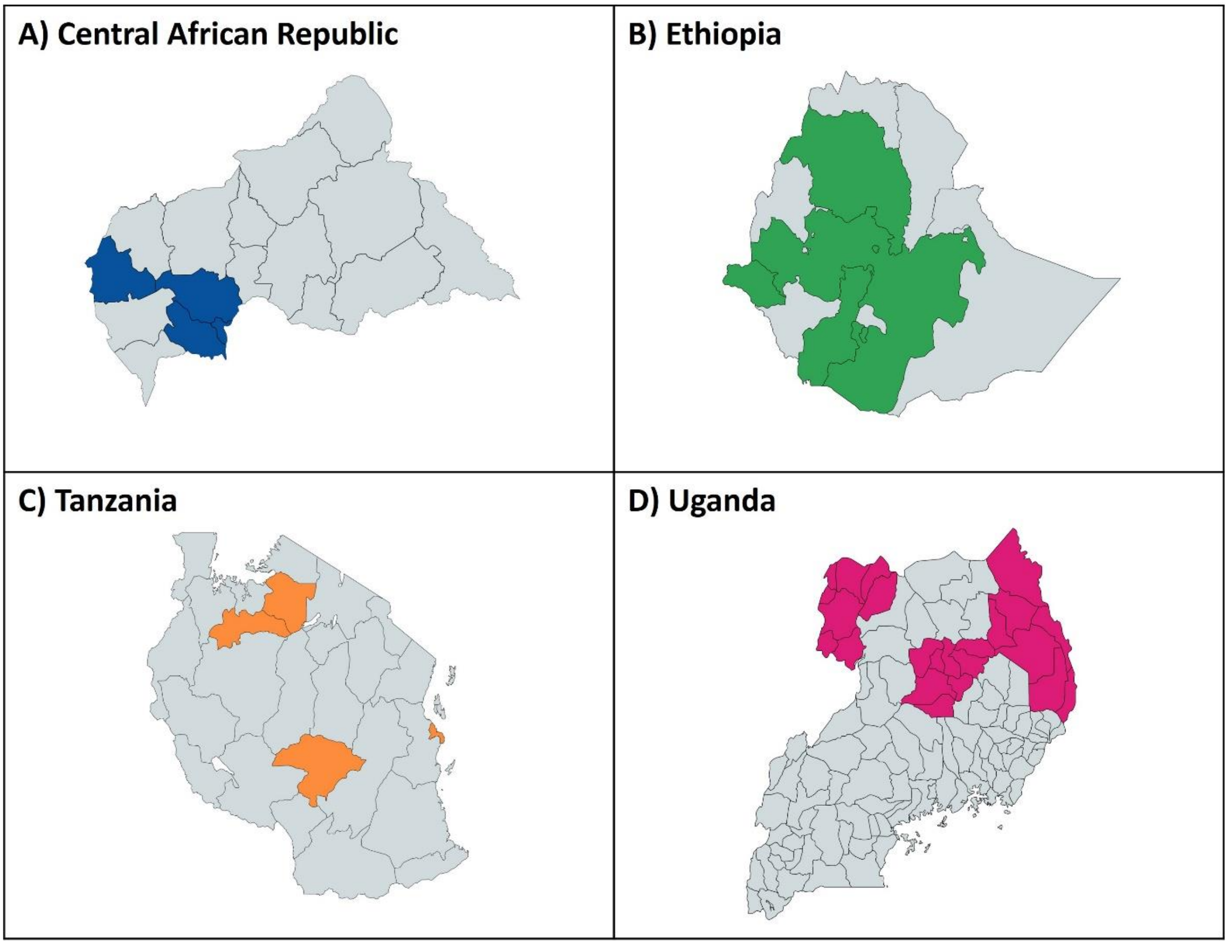

Notes: the figure shows regions included in the IMPULSE study. For Central African Republic: Bangui City Administration and Health region 1, 2 and 7; for Ethiopia: Addis Ababa City Administration, Oromia, Amhara and Gambella, South Ethiopia and Sidama; for Tanzania: Dar es Salaam City Administration, Iringa, Shinyanga, Simiyu; for Uganda: Lango, Karamoja, West-Nile, Kampala City Administration.

Appendix S4. Characteritics of the regions

	CAR					Ethiopia					Tanzania					Uganda					
**Key characteritics**	Bangui City Administration	Health region 1	Health region 2	Health region 7	Total CAR	Addis Ababa City Administration	Oromia	**Amhara and Gambella**	South Ethiopia and Sidama	Total Ethiopia	Dar es Salaam City Administration	Iringa	Shinyanga	Simiyu	Total Tanzania	Kampala City Administration	**Lango**	Karamoja	West-Nile	Total Uganda	TOTAL
Setting																					
Urban	Yes				1	Yes				1	Yes				1	Yes				1	4
Rural			Yes		1			Yes	Yes	2			Yes	Yes	2			Yes		1	6
Mixed		Yes		Yes	2		Yes			1		Yes			1		Yes		Yes	2	6
Hard to reach / disadvantaged area									Yes (semi nomadic population, fload risk)	1			Yes	Yes	2			Yes (semi nomadic population)		1	4
Humanitarian setting	Yes (conflict)	Yes (conflict)	Yes (conflict)	Yes (conflict)	4			Yes (Gambella: refugees)		1									yes (refugees)	1	6

Abbreviations: CAR = Central African Republic

Appendix S5. Sampling criteria

	Criteria
Health facilities	
3rd level of referral and/or National	1
In each region:	
3rd level of referral (Regional)	1
2nd level of referral (Subnational / District) Public	2
2nd level of referral (Subnational / District) Private Not For Profit *	1
2nd level of referral (Subnational / District) Private for Profit *	1
1st level of referral (Primary Hospital / Health Center with CEmONC) Public	3
1st level of referral (Primary Hospital / Health Center with CEmONC) Private Not For Profit *	1
1st level of referral (Primary Hospital / Health Center with CEmONC) Private for Profit *	1-2
Data offices	
District /Subnational Health Office	all data offices related to selected facilities
Regional Health office	yes
Central Ministry of Health	yes

Notes: Only CEmONC facilities except for CAR where BEmONC facilities were included; * if existing and allowing.

Abbreviations: BEmONC= basic Emergency Obstetric and Neonatal Care; CEmONC= Comprehensive Emergency Obstetric and Newborn Care

Appendix S6. Formulas for the composite indicators according to the PRISM User's Kit 2019

Average score on data quality control

Indicators:

- **Average score on data quality control**

> This indicator is composed of multiple questions.
>
> If the respondent answers anything other than the answer equated with code "1" on any of the questions included in the numerator calculation, their answer is not counted in the numerator.
>
> The maximum score that can be attained (which appears in the denominator) is 8 (equivalent to 8 "yes" answers) at the district level, and 7 (equivalent to 7 "yes" answers) at the health facility level.

$$\% = 100 \text{ x } \frac{\text{Sum of the district's data quality control score}}{\text{Total \# of districts assessed x 8}}$$

Data Source: Module 2a. RHIS Performance Diagnostic Tool (District Level)		
Indicator	Numerator	Denominator
District data quality score	Sum of **DQ011**=1<br>+ Sum of **DQ12b**=1<br>+ Sum of **DQ013b**=1<br>+ Sum of **DQ029**=1<br>+ Sum of **DQ030**=1<br>+ Sum of **DQ031**=1<br>+ Sum of **DQ032**=1<br>+ Sum of **DQ033**=1	8 x number of districts assessed

$$\% = 100 \text{ x } \frac{\text{Sum of the facility's data quality control score}}{\text{Total \# of facilities assessed x 7}}$$

Data Source: Module 2b. RHIS Performance Diagnostic Tool (Health Facility Level)		
Indicator	Numerator	Denominator
Facility data quality score	Sum of **FQ012**=1<br>+ Sum of **FQ013b**=1<br>+ Sum of **FQ063**=1<br>+ Sum of **FQ064**=1<br>+ Sum of **FQ065**=1<br>+ Sum of **FQ066**=1<br>+ Sum of **FQ067**=1	7 x number of facilities assessed

Average score for quality of supervision

- **Average score for quality of supervision**

$$\% = 100 \text{ x } \frac{\text{Sum of the facility's points}}{\text{Total \# of facilities supervised x 5}}$$

The method to calculate a facility's score is to add the number of points based on the respondent's answers. These points are your numerator. Numerator scores can range from 1 to 5 for each site.

Data Source: Module 2b. RHIS Performance Diagnostic Tool (Health Facility Level)		
Indicator	Points to add to numerator	Denominator
Overall quality of supervision	1 point if sum of **FU023**=1<br>+ 1 point if sum of **FU024**=1<br>+ 1 point if sum of **FU025**=1<br>+ 1 point if sum of **FU026**=1<br>+ 1 point if sum of **FU027**=1	5 x [Count of **FU022**=1<br>+ Count of **FU022**=2<br>+ Count of **FU022**=3<br>+ Count of **FU022**=4<br>+ Count of **FU022**=5]

Reference: MEASURE Evaluation. Performance of Routine Information System Management (PRISM) User's Kit: Preparing and Conducting a PRISM Assessment — MEASURE Evaluation. 2018. Available: https://www.measureevaluation.org/resources/publications/ms-18-140.html

Abbreviations: PRISM = Performance of Routine Information System Management

Appendix S7. Sample characteristics by IMPULSE administrative unit and regions

EN-MINI-PRISM Tool	N of analysed measures	Sample characteristics		Overall n	Overall %	CAR n	CAR %	Ethiopia n	Ethiopia %	Tanzania n	Tanzania %	Uganda n	Uganda %
Tool 4	23			N=56		N=7		N=11		N=17		N=21	
		Data office characteristics	District health data office	45	80.4	6	85.7	6	54.5	13	76.5	20	95.2
			Central/regional health data office	11	19.6	1	14.3	5	45.5	4	23.5	1	4.8
		Regions	**CAR**	**7**	**12.5**								
			Bangui City Administration	1	1.8	1	14.3	-	-	-	-	-	-
			Health region 1	1	1.8	1	14.3	-	-	-	-	-	-
			Health region 2	2	3.6	2	28.6	-	-	-	-	-	-
			Health region 7	3	5.4	3	42.9	-	-	-	-	-	-
			**Ethiopia**	**11**	**19.6**								
			Addis Ababa City Administration	2	3.6	-	-	2	18.2	-	-	-	-
			Oromia	3	5.4	-	-	3	27.3	-	-	-	-
			Amhara and Gambella	2	3.6	-	-	2	18.2	-	-	-	-
			South Ethiopia and Sidama	4	7.1	-	-	4	36.4	-	-	-	-
			**Tanzania**	**16**	**28.6**								
			Dar es Salaam City Administration	1	1.8	-	-	-	-	1	5.9	-	-
			Iringa	6	10.7	-	-	-	-	6	35.3	-	-
			Shinyanga	4	7.1	-	-	-	-	4	23.5	-	-
			Simiyu	6	10.7	-	-	-	-	6	35.3	-	-
			**Uganda**	**21**	**37.5**								
			Kampala City Administration	1	1.8	-	-	-	-	-	-	1	4.8
			Lango	6	10.7	-	-	-	-	-	-	6	28.6
			Karamoja	8	14.3	-	-	-	-	-	-	8	38.1
			West-Nile	6	10.7	-	-	-	-	-	-	6	28.6
		Managing authority	Public	56	100	7	100	11	100	17	100	21	100
		End-users		N=56		N=7		N=11		N=17		N=21	
			End-users from district health data office	45	80.4	6	85.7	6	54.5	13	76.5	20	95.2
			End-users from central/regional health data office	11	19.6	1	14.3	5	45.5	4	23.5	1	4.8

EN-MINI-PRISM Tool	N of analysed measures	Sample characteristics		Overall n	Overall %	CAR n	CAR %	Ethiopia n	Ethiopia %	Tanzania n	Tanzania %	Uganda n	Uganda %
Tool 2a	10			N=49		N=6		N=6		N=17		N=20	
		Data office characteristics	District health data office	46	93.9	6	100	6	100	14	82.4	20	100
			Central/regional health data office	3	6.1	0	0	0	0	3	17.6	0	0
		Regions	**CAR**	**6**	**12.2**								
			Health region 1	1	2.0	1	16.7	-	-	-	-	-	-
			Health region 2	2	4.1	2	33.3	-	-	-	-	-	-
			Health region 7	3	6.1	3	50.0	-	-	-	-	-	-
			Bangui City Administration	0	0.0	0	0.0	-	-	-	-	-	-
			**Ethiopia**	**6**	**12.2**								
			Addis Ababa City Administration	0	0.0	-	-	0	0.0	-	-	-	-
			Oromia	2	4.1	-	-	2	33.3	-	-	-	-
			Amhara and Gambella	1	2.0	-	-	1	16.7	-	-	-	-
			South Ethiopia and Sidama	3	6.1	-	-	3	50.0	-	-	-	-
			**Tanzania**	**17**	**34.7**								
			Iringa	6	12.2	-	-	-	-	6	35.3	-	-
			Shinyanga	5	10.2	-	-	-	-	5	29.4	-	-
			Simiyu	6	12.2	-	-	-	-	6	35.3	-	-

			**Uganda**	**20**	**40.8**	-	-	-	-	-	-		
			Kampala City Administration	0	0.0	-	-	-	-	-	-	0	0.0
			Lango	6	12.2	-	-	-	-	-	-	6	30.0
			Karamoja	8	16.3	-	-	-	-	-	-	8	40.0
			West-Nile	6	12.2	-	-	-	-	-	-	6	30.0
		Managing authority	Public	49	100	6	100	6	100	17	100	20	100
Tool 2b	19			N=95		N=14		N=24		N=29		N=28	
		Health facilities level	Third level of referral	17	17.9	4	28.6	3	12.5	5	17.2	5	17.9
			Second level of referral	39	41.1	3	21.4	10	41.7	15	51.7	11	39.3
			First level of referral	39	41.1	7	50	11	45.8	9	31	12	42.9
		Regions	**CAR**	**14**	**14.7**								
			Health region 1	3	3.2	3	21.4	-	-	-	-	-	-
			Health region 2	4	4.2	4	28.6	-	-	-	-	-	-
			Health region 7	4	4.2	4	28.6	-	-	-	-	-	-
			Bangui City Administration	3	3.2	3	21.4	-	-	-	-	-	-
			**Ethiopia**	**24**	**25.3**								
			Addis Ababa City Administration	3	3.2	-	-	3	12.5	-	-	-	-
			Oromia	9	9.5	-	-	9	37.5	-	-	-	-
			Amhara and Gambella	3	3.2	-	-	3	12.5	-	-	-	-
			South Ethiopia and Sidama	9	9.5	-	-	9	37.5	-	-	-	-
			**Tanzania**	**27**	**28.4**								
			Dar es Salaam City Administration	2	2.1	-	-	-	-	2	6.9	-	-
			Iringa	10	10.5	-	-	-	-	10	34.5	-	-
			Shinyanga	9	9.5	-	-	-	-	9	31.0	-	-
			Simiyu	8	8.4	-	-	-	-	8	27.6	-	-
			**Uganda**	**28**	**29.5**								
			Kampala City Administration	1	1.1	-	-	-	-	-	-	1	3.6
			Lango	9	9.5	-	-	-	-	-	-	9	32.1
			Karamoja	7	7.4	-	-	-	-	-	-	7	25.0
			West-Nile	11	11.6	-	-	-	-	-	-	11	39.3
		Urban/Rural	Rural	43	45.3	1	7.1	6	25	21	72.4	15	53.6
			Urban	52	54.7	13	92.9	18	75	8	27.6	13	46.4
		Managing authority	Public	74	77.9	12	85.7	18	75	22	75.9	22	78.6
			Not for profit	12	12.6	1	7.1	1	4.2	4	13.8	6	21.4
			Private	9	9.5	1	7.1	5	20.8	3	10.3	0	0
		End-users		N=52		N=0		N=10		N=24		N=18	
			End-users from third level of referral	8	15.4			2	20.0	4	16.7	2	11.1
			End-users from second level of referral	23	44.2			3	30.0	11	45.8	9	50.0
			End-users from first level of referral	21	40.4			5	50.0	9	37.5	7	38.9

Abbreviations: CAR = Central African Republic

Appendix S8. Governance, planning, financing, capacity development at data office level *

Variable identifier in EN-MINI tools			Overall N=56*		CAR										Ethiopia										Tanzania										Uganda										p-value §
					Bangui City Administration N=1		Health region 1 N=1		Health region 2 N=2		Health region 7 N=3		Total CAR N=7		Addis Ababa City Administration N=2		Oromia N=3		Amhara and Gambella N=2		South Ethiopia and Sidama N=4		Total Ethiopia N=11		Dar es Salaam City Administration N=1		Iringa N=6		Shinyanga N=4		Simiyu N=6		Total Tanzania N=17		Kampala City Administration N=1		Lango N=6		Karamoja N=8		West-Nile N=6		Total Uganda N=21		
		Answer	n	%	n	%	n	%	n	%	n	%	n	%	n	%	n	%	n	%	n	%	n	%	n	%	n	%	n	%	n	%	n	%	n	%	n	%	n	%	n	%	n	%	
	Governance																																												
MAT005	Written RHIS missions, roles and responsibilities	Yes	46	82.1	1	100	0	0	2	100	2	66.7	5	71.4	2	100	2	66.7	1	50	4	100	9	81.8	1	100	6	100	4	100	5	83.3	16	94.1	1	100	4	66.7	6	75	5	83.3	16	76.2	0.380
MAT006	Organization charts showing health information positions	Yes	41	73.2	1	100	0	0	1	50	2	66.7	4	57.1	2	100	3	100	1	50	4	100	10	90.9	1	100	5	83.3	2	50	2	33.3	10	58.8	1	100	5	83.3	6	75	5	83.3	17	81	0.156
MAT007A	Written SOP and guidelines □	Yes	31	55.4	0	0	0	0	0	0	0	0	0	0	2	100	3	100	1	50	3	75	9	81.8	1	100	1	16.7	3	75	5	83.3	10	58.8	0	0	5	83.3	3	37.5	4	66.7	12	57.1	0.006
		Yes, partially	15	26.8	1	100	0	0	0	0	0	0	1	14.3	0	0	0	0	0	0	1	25	1	9.1	0	0	4	66.7	0	0	1	16.7	5	29.4	1	100	0	0	5	62.5	2	33.3	8	38.1	0.354
MAT008	Information and communication technology plan	Yes	26	46.4	1	100	0	0	0	0	0	0	1	14.3	2	100	3	100	1	50	4	100	10	90.9	1	100	1	16.7	3	75	3	50	8	47.1	1	100	1	16.7	3	37.5	2	33.3	7	33.3	0.003
MAT009	List/documentation of RHIS report disseminations	Yes	40	71.4	1	100	0	0	0	0	0	0	1	14.3	1	50	3	100	2	100	4	100	10	90.9	1	100	4	66.7	2	50	6	100	13	76.5	1	100	5	83.3	5	62.5	5	83.3	16	76.2	0.006
	Planning																																												
MAT-010	Copy of national RHIS analysis/assessment report (less than three years old)	Yes	21	37.5	0	0	0	0	0	0	0	0	0	0	1	50	2	66.7	1	50	3	75	7	63.6	1	100	3	50	1	25	1	16.7	6	35.3	1	100	3	50	3	37.5	1	16.7	8	38.1	0.078
MAT-011	Copy of national RHIS strategic plan	Yes	34	60.7	1	100	0	0	0	0	0	0	1	14.3	2	100	3	100	2	100	4	100	11	100	1	100	3	50	3	75	2	33.3	9	52.9	1	100	4	66.7	5	62.5	3	50	13	61.9	0.001
MAT-012.1	Performance targets for data accuracy	Yes	40	71.4	0	0	0	0	0	0	0	0	0	0	1	50	3	100	2	100	4	100	10	90.9	1	100	3	50	4	100	5	83.3	13	76.5	1	100	4	66.7	7	87.5	5	83.3	17	81	<0.001
MAT-012.2	Performance targets for data completeness	Yes	43	76.8	0	0	0	0	0	0	0	0	0	0	2	100	3	100	2	100	4	100	11	100	1	100	2	33.3	4	100	5	83.3	12	70.6	1	100	6	100	8	100	5	83.3	20	95.2	<0.001
MAT-012.3	Performance targets for data timeliness	Yes	45	80.4	0	0	0	0	0	0	0	0	0	0	2	100	3	100	2	100	4	100	11	100	1	100	3	50	4	100	5	83.3	13	76.5	1	100	6	100	8	100	6	100	21	100	<0.001
	Financing																																												
MAT-022	Budget for RHIS supplies	Yes	14	25	0	0	0	0	0	0	0	0	0	0	2	100	1	33.3	0	0	0	0	3	27.3	1	100	1	16.7	2	50	2	33.3	6	35.3	1	100	2	33.3	1	12.5	1	16.7	5	23.8	0.079
MAT-023	Access to financial and logistics resources	Yes	28	50	0	0	0	0	0	0	1	33.3	1	14.3	2	100	1	33.3	1	50	0	0	4	36.4	1	100	2	33.3	1	25	2	33.3	6	35.3	1	100	5	83.3	6	75	5	83.3	17	81	0.002
MAT-024	Copy of long-term financial plan	Yes	20	35.7	0	0	0	0	0	0	0	0	0	0	2	100	0	0	0	0	3	75	5	45.5	1	100	2	33.3	0	0	2	33.3	5	29.4	1	100	3	50	5	62.5	1	16.7	10	47.6	0.104
	Capacity development																																												
MAT-013	Copy of training need assessment	Yes	15	26.8	0	0	0	0	0	0	0	0	0	0	1	50	1	33.3	1	50	0	0	3	27.3	0	0	1	16.7	3	75	3	50	7	41.2	0	0	2	33.3	2	25	1	16.7	5	23.8	0.333
MAT-014	RHIS training manual	Yes	36	64.3	1	100	0	0	0	0	1	33.3	2	28.6	2	100	3	100	2	100	4	100	11	100	1	100	3	50	3	75	3	50	10	58.8	1	100	5	83.3	4	50	3	50	13	61.9	0.01
MAT-015	Training using the RHIS manual	Yes	30	53.6	1	100	0	0	0	0	0	0	1	14.3	2	100	3	100	2	100	4	100	11	100	1	100	1	16.7	1	25	2	33.3	5	29.4	1	100	5	83.3	4	50	3	50	13	61.9	0.001
MAT-016	Costed training and capacity development plan	Yes	29	51.8	0	0	0	0	0	0	1	33.3	1	14.3	2	100	3	100	1	50	2	50	8	72.7	1	100	1	16.7	1	25	5	83.3	8	47.1	1	100	6	100	3	37.5	2	33.3	12	57.1	0.106
MAT-017	Schedule for training	Yes	17	30.4	1	100	0	0	0	0	1	33.3	2	28.6	2	100	1	33.3	0	0	0	0	3	27.3	0	0	0	0	2	50	1	16.7	3	17.6	1	100	5	83.3	2	25	1	16.7	9	42.9	0.412
	Areas where SOP are lacking ◊		N=15		N=1		N=0		N=0		N=0		N=1		N=0		N=0		N=0		N=1		N=1		N=0		N=4		N=0		N=1		N=5		N=1		N=0		N=5		N=2		N=8		
MAT007b	1. Data definitions	Yes	2	13.3	1	100							1	100							0	0	0	0			0	0			1	100	1	20	0	100			0	0	0	0	0	0	0.105
MAT007b	2. Data collection and reporting	Yes	5	33.3	1	100							1	100							0	0	0	0			3	75			1	100	4	80	0	0			0	0	0	0	0	0	0.004
MAT007b	3. Data aggregation, processing, transmission	Yes	6	40	1	100							1	100							0	0	0	0			3	75			1	100	4	80	0	0			1	20	0	0	1	12.5	0.025
MAT007b	4. Data analysis, dissemination, use	Yes	7	46.7	0	0							0	0							0	0	0	0			4	100			1	100	5	100	0	0			1	20	1	50	2	25	0.017
MAT007b	5. Data quality assurance	Yes	4	26.7	0	0							0	0							0	0	0	0			2	50			1	100	3	60	0	0			1	20	0	0	1	12.5	0.385
MAT007b	6. Master facility list	Yes	7	46.7	1	100							1	100							0	0	0	0			1	25			0	0	1	20	0	0			3	60	2	100	5	62.5	0.271
MAT007b	7. ICD codes	Yes	7	46.7	0	0							0	0							1	100	1	100			1	25			0	0	1	20	0	0			3	60	2	100	5	62.5	0.271
MAT007b	8. Data security	Yes	9	60	0	0							0	0							1	100	1	100			1	25			1	100	2	40	1	100			3	60	2	100	6	75	0.385
MAT007b	9. Data storage	Yes	5	33.3	1	100							1	100							0	0	0	0			2	50			1	100	3	60	0	0			1	20	0	0	1	12.5	0.184
MAT007b	10. Performance improvement processes	Yes	5	33.3	1	100							1	100							0	0	0	0			2	50			1	100	3	60	0	0			0	0	1	50	1	12.5	0.184

Notes: * all data from Tool 4;

□ written SOP and procedural guidelines for RHIS that include:

- Data definitions including newborn and stillbirth data elements/ indicators
- Data collection and reporting including newborn and stillbirth data elements/ indicators
- Data aggregation, processing, and transmission including newborn and stillbirth data elements/ indicators
- Data analysis, dissemination, and use including newborn and stillbirth data elements/ indicators
- Data quality assurance including newborn and stillbirth data elements/ indicators
- Master facility list (MFL)
- International Classification of Diseases (ICD) codes relevant to newborns and stillbirths
- Data security
- Data storage
- Performance improvement processes

◊ Data on areas where SOP are lacking were collected for the subset of data offices evaluating that written SOP and guidelines were partially present;
§ comparison of frequencies across countries.

Abbreviations: CAR = Central African Republic; EN-MINI Tools= Every Newborn-Measurement Improvement for Newborn & Stillbirth Measures Tools; ICD = International classification of diseases; RHIS = routine health information system; SOP = standard operating procedures.

Appendix S9. Guidelines, data quality assurance systems and feedback mechanisms

					CAR										Ethiopia										Tanzania										Uganda										
Variable identifier in EN-MINI tools		Answer	Overall		Bangui City Administration		Health region 1		Health region 2		Health region 7		Total CAR		Addis Abeba City Administration		Oromia		Amhara and Gambella		South Ethiopia and Sidama		Total Ethiopia		Dar es Salaam City Administration		Iringa		Shinyanga		Simiyu		Total Tanzania		Kampala City Administration		Lango		Karamoja		West-Nile		Total Uganda		p-value §
			n	%	n	%	n	%	n	%	n	%	n	%	n	%	n	%	n	%	n	%	n	%	n	%	n	%	n	%	n	%	n	%	n	%	n	%	n	%	n	%	n	%	
	Guidelines																																												
	Data office †		N=49		N=0		N=1		N=2		N=3		N=6		N=0		N=2		N=1		N=3		N=6		N=0		N=6		N=5		N=6		N=17		N=0		N=6		N=8		N=6		N=20		
DQ_012a	Written guidelines for data entry/compilation	Yes	40	81.6			0	0	0	0	0	0	0	0			2	100	1	100	3	100	6	100			5	83.3	5	100	6	100	16	94.1			5	83.3	7	87.5	6	100	18	90	<0.001
DQ_012b	Written guidelines for data review and quality control*	Yes	39	79.6			0	0	0	0	0	0	0	0			2	100	1	100	3	100	6	100			5	83.3	5	100	6	100	16	94.1			4	66.7	7	87.5	6	100	17	85	<0.001
DQ_029	Written guidelines on routine health data quality assessments/assurance	Yes	36	73.5			0	0	0	0	0	0	0	0			2	100	1	100	3	100	6	100			3	50	4	80	6	100	13	76.5			5	83.3	7	87.5	5	83.3	17	85	<0.001
	Health facility ‡		N=95		N=3		N=3		N=4		N=4		N=14		N=3		N=9		N=3		N=9		N=24		N=2		N=10		N=9		N=8		N=29		N=1		N=9		N=7		N=11		N=28		
FQ_015.1	Guidelines including what they are supposed to report on	Yes/Mostly	68	71.6	2	66.7	3	100	0	0	2	50	7	50	3	100	3	33.3	1	33.3	4	44.4	11	45.8	2	100	10	100	7	77.8	7	87.5	26	89.7	1	100	6	66.7	7	100	10	90.9	24	85.7	<0.001
FQ_015.2	Guidelines including how reports are to be submitted	Yes/Mostly	67	70.5	2	66.7	3	100	0	0	2	50	7	50	3	100	3	33.3	1	33.3	4	44.4	11	45.8	2	100	10	100	7	77.8	7	87.5	26	89.7	1	100	6	66.7	6	85.7	10	90.9	23	82.1	0.001
FQ_015.3	Guidelines including to whom the reports should be submitted	Yes/Mostly	68	71.6	2	66.7	3	100	0	0	2	50	7	50	3	100	3	33.3	1	33.3	5	55.6	12	50	2	100	10	100	7	77.8	7	87.5	26	89.7	1	100	6	66.7	6	85.7	10	90.9	23	82.1	0.002
FQ_015.4	Guidelines including when the reports are due	Yes/Mostly	69	72.6	2	66.7	3	100	0	0	2	50	7	50	3	100	3	33.3	1	33.3	5	55.6	12	50	2	100	10	100	7	77.8	7	87.5	26	89.7	1	100	7	77.8	6	85.7	10	90.9	24	85.7	0.001
FQ_063	Written instructions/guidelines on how to perform a data quality review or data quality check*	Yes	53	55.8	0	0	0	0	1	25	0	0	1	7.1	3	100	7	77.8	3	100	6	66.7	19	79.2	1	50	4	40	7	77.8	4	50	16	55.2	1	100	5	55.6	6	85.7	5	45.5	17	60.7	<0.001
	Data quality assurance system in place																																												
	Data office †		N=49		N=0		N=1		N=2		N=3		N=6		N=0		N=2		N=1		N=3		N=6		N=0		N=6		N=5		N=6		N=17		N=0		N=6		N=8		N=6		N=20		
DQ_011	Designated staff for internal data quality review*	Yes	48	98			1	100	2	100	3	100	6	100			1	50	1	100	3	100	5	83.3			6	100	5	100	6	100	17	100			6	100	8	100	6	100	20	100	0.245
DQ_013b	Designated staff trained in data review and quality control*	Yes	24	49			1	100	0	0	1	33.3	2	33.3			1	50	0	0	2	66.7	3	50			1	16.7	3	60	1	16.7	5	29.4			4	66.7	6	75	4	66.7	14	70	0.075
DQ_030	Data quality assessments*	Yes	42	85.7			0	0	0	0	1	33.3	1	16.7			2	100	1	100	3	100	6	100			4	66.7	5	100	6	100	15	88.2			6	100	8	100	6	100	20	100	<0.001
DQ_031	Use of data quality assessment tools/in-built electronic data quality validation rules/system*	Yes	38	77.6			0	0	0	0	0	0	0	0			2	100	1	100	3	100	6	100			4	66.7	3	60	6	100	13	76.5			5	83.3	8	100	6	100	19	95	0.102
DQ_032	Record of health facility data quality assessments conducted in the past 12 months*	Yes	40	81.6			0	0	0	0	0	0	0	0			2	100	1	100	3	100	6	100			4	66.7	5	100	6	100	15	88.2			5	83.3	8	100	6	100	19	95	0.065
	Health facility ‡		N=95		N=3		N=3		N=4		N=4		N=14		N=3		N=9		N=3		N=9		N=24		N=2		N=10		N=9		N=8		N=29		N=1		N=9		N=7		N=11		N=28		
FQ_012	Designated staff for internal data quality review*	Yes	76	80	3	100	2	66.7	2	50	1	25	8	57.1	3	100	8	88.9	3	100	6	66.7	20	83.3	0	0	9	90	8	88.9	5	62.5	22	75.9	1	100	9	100	7	100	9	81.8	26	92.9	0.05
FQ_013a	Designated staff trained in data entry/compilation	Yes	37	38.9	0	0	0	0	0	0	0	0	0	0	2	66.7	6	66.7	1	33.3	3	33.3	12	50	0	0	3	30	2	22.2	1	12.5	6	20.7	1	100	4	44.4	5	71.4	9	81.8	19	67.9	<0.001
FQ_013b	Designated staff trained in data review and quality control*	Yes	33	34.7	0	0	0	0	0	0	0	0	0	0	2	66.7	1	11.1	0	0	3	33.3	6	25	0	0	3	30	3	33.3	1	12.5	7	24.1	1	100	4	44.4	5	71.4	10	90.9	20	71.4	<0.001
FQ_064	Regular data accuracy checks (data quality self-assessment)*	Yes	67	70.5	1	33.3	0	0	0	0	1	25	2	14.3	3	100	6	66.7	3	100	7	77.8	19	79.2	1	50	6	60	9	100	8	100	24	82.8	1	100	6	66.7	6	85.7	9	81.8	22	78.6	<0.001
FQ_065	Access to data quality self-assessment tools (paper or electronic)*	Yes	54	56.8	0	0	0	0	0	0	0	0	0	0	3	100	6	66.7	3	100	6	66.7	18	75	1	50	1	10	8	88.9	8	100	18	62.1	1	100	4	44.4	6	85.7	7	63.6	18	64.3	0.022
FQ_066	Record of health facility data accuracy self-assessments conducted in the past three months*	Yes	48	50.5	0	0	0	0	0	0	0	0	0	0	3	100	6	66.7	3	100	6	66.7	18	75	1	50	4	40	4	44.4	4	50	13	44.8	1	100	3	33.3	5	71.4	8	72.7	17	60.7	0.002
	Feedback Mechanism in Place																																												
	Data office †		N=49		N=0		N=1		N=2		N=3		N=6		N=0		N=2		N=1		N=3		N=6		N=0		N=6		N=5		N=6		N=17		N=0		N=6		N=8		N=6		N=20		
DQ_033	Feedback records to health facilities on data quality assessments *	Yes	37	75.5			0	0	0	0	0	0	0	0			2	100	1	100	3	100	6	100			4	66.7	5	100	6	100	15	88.2			5	83.3	8	100	3	50	16	80	0.033
DU_009	District office sent feedback reports to facilities in the last 3 months	Yes	36	73.5			0	0	0	0	1	33.3	1	16.7			2	100	1	100	3	100	6	100			5	83.3	4	80	6	100	15	88.2			5	83.3	5	62.5	4	66.7	14	70	0.004

	**Health facility ‡**		**N=95**	N=3	N=3	N=4	N=4	**N=14**	N=3	N=9	**N=3**	N=9	**N=24**	**N=2**	N=10	N=9	N=8	**N=29**	N=1	**N=9**	N=7	N=11	**N=28**	
FQ_067	Feedback records to staff on data quality*	Yes	49 51.6	0 0	0 0	0 0	0 0	0 0	3 100	6 66.7	3 100	5 55.6	17 70.8	1 50	4 40	7 77.8	3 37.5	15 51.7	1 100	4 44.4	5 71.4	7 63.6	17 60.7	0.026
FU_009	Feedback reports from the district office/MOH in the last 3 months	Yes	46 48.4	0 0	0 0	0 0	0 0	0 0	3 100	5 55.6	1 33.3	2 22.2	11 45.8	1 50	5 50	6 66.7	8 100	20 69	1 100	3 33.3	4 57.1	7 63.6	15 53.6	<0.001
	**PRISM average score on data quality control □**																							
	**Data office †**		**N=49**	N=0	N=1	N=2	N=3	**N=6**	N=0	N=2	**N=1**	N=3	**N=6**	**N=0**	N=6	N=5	N=6	**N=17**	N=0	**N=6**	N=8	N=6	**N=20**	
	Data office data quality control score (%) □		77.55		25	12.5	20.83	18.75		87.5	87.5	95.83	91.67		64.58	87.5	89.58	80.15		83.33	93.75	87.5	88.75	
	**Health facility ‡**		**N=95**	N=3	N=3	N=4	N=4	**N=14**	N=3	N=9	**N=3**	N=9	**N=24**	**N=2**	N=10	N=9	N=8	**N=29**	N=1	**N=9**	N=7	N=11	**N=28**	
	Health facility data quality control score (%) □		57.14	19.05	9.52	10.71	7.14	11.22	95.24	63.49	85.71	61.9	69.64	35.71	44.29	73.02	58.93	56.65	100	55.56	81.63	71.43	69.9	

Notes: † from Tool 2a; ‡ from Tool 2b; * variables contributing to the data quality score; □ data quality score calculated according to the PRISM user's kit, formulas are reported in Appendix S6; § comparison of frequencies across countries.

Abbreviations: CAR = Central African Republic; EN-MINI Tools= Every Newborn-Measurement Improvement for Newborn & Stillbirth Measures Tools; MOH = ministry of health; PRISM = Performance of Routine Information System Management; RHIS = routine health information systems

Appendix S10. Supportive supervision from data office

Variable identifier in EN-MINI tools		Answer	Overall		CAR										Ethiopia										Tanzania										Uganda										p-value §
					Bangui City Administration		Health region 1		Health region 2		Health region 7		**Total CAR**		Addis Abeba City Administration		Oromia		Amhara and Gambella		South Ethiopia and Sidama		**Total Ethiopia**		Dar es Salaam City Administration		Iringa		Shinyanga		Simiyu		**Total Tanzania**		Kampala City Administration		Lango		Karamoja		West-Nile		**Total Uganda**		
			n	%	n	%	n	%	n	%	n	%	n	%	n	%	n	%	n	%	n	%	n	%	n	%	n	%	n	%	n	%	n	%	n	%	n	%	n	%	n	%	n	%	
	**Supportive supervision***		**N=56 ***		N=1		N=1		N=2		N=3		**N=7**		N=2		N=3		N=2		N=4		**N=11**		N=1		N=6		N=4		N=6		**N=17**		N=1		N=6		N=8		N=6		**N=21**		
MAT-018	Copy of supervisory guidelines available	Yes	27	48.2	0	0	0	0	0	0	0	0	0	0	1	50	3	100	2	100	4	100	10	90.9	1	100	4	66.7	1	25	4	66.7	10	58.8	0	0	3	50	4	50	0	0	7	33.3	<0.001
MAT-019	Regular schedule of RHIS supervision maintained	Yes	45	80.4	0	0	0	0	0	0	1	33.3	1	14.3	2	100	3	100	2	100	4	100	11	100	1	100	4	66.7	4	100	6	100	15	88.2	0	0	6	100	8	100	4	66.7	18	85.7	<0.001
MAT-020	Copy of the RHIS supervisory visit reports available	Yes	41	73.2	0	0	0	0	0	0	1	33.3	1	14.3	1	50	3	100	2	100	4	100	10	90.9	1	100	3	50	4	100	6	100	14	82.4	1	100	5	83.3	8	100	2	33.3	16	76.2	0.004
MAT-021	Facility supervised received a copy of the report	Yes	39	69.6	0	0	0	0	0	0	0	0	0	0	2	100	3	100	2	100	4	100	11	100	1	100	3	50	4	100	6	100	14	82.4	0	0	4	66.7	6	75	4	66.7	14	66.7	<0.001
	**Supervision quality ‡**		**N=95 ‡**		N=3		N=3		N=4		N=4		**N=14**		N=3		N=9		N=3		N=9		**N=24**		N=2		N=10		N=9		N=8		**N=29**		N=1		N=9		N=7		N=11		**N=28**		
FU022	District supervisory visits in the last 3 months:																																										
	One or more		52	54.7	0	0	0	0	0	0	0	0	0	0	3	100	4	44.4	0	0	3	33.3	10	41.7	1	50	7	70	8	88.9	8	100	24	82.8	1	100	6	66.7	5	71.4	6	54.5	18	64.3	<0.001
	More than four times		11	11.6	0	0	0	0	0	0	0	0	0	0	0	0	0	0	0	0	0	0	0	0	0	0	2	20	3	33.3	2	25	7	24.1	1	100	0	0	2	28.6	1	9.1	4	14.3	
	Four times		2	2.1	0	0	0	0	0	0	0	0	0	0	0	0	0	0	0	0	0	0	0	0	0	0	0	0	1	11.1	0	0	1	3.4	0	0	0	0	0	0	1	9.1	1	3.6	
	Three times		11	11.6	0	0	0	0	0	0	0	0	0	0	0	0	1	11.1	0	0	0	0	1	4.2	0	0	1	10	0	0	2	25	3	10.3	0	0	3	33.3	1	14.3	3	27.3	7	25	
	Two times		7	7.4	0	0	0	0	0	0	0	0	0	0	1	33.3	0	0	0	0	0	0	1	4.2	0	0	1	10	2	22.2	3	37.5	6	20.7	0	0	0	0	0	0	0	0	0	0	
	One time		21	22.1	0	0	0	0	0	0	0	0	0	0	2	66.7	3	33.3	0	0	3	33.3	8	33.3	1	50	3	30	2	22.2	1	12.5	7	24.1	0	0	3	33.3	2	28.6	1	9.1	6	21.4	
	None		43	45.3	3	100	4	100	4	100	3	100	14	100	0	0	5	55.6	3	100	6	66.7	14	58.3	1	50	3	30	1	11.1	0	0	5	17.2	0	0	3	33.3	2	28.6	5	45.5	10	35.7	
			**N=52**		N=0		N=0		N=0		N=0		**N=0**		N=3		N=4		N=0		N=3		**N=10**		N=1		N=7		N=8		N=8		**N=24**		N=1		N=6		N=5		N=6		**N=18**		
FU023	District supervisor checked the newborn and stillbirth data quality ◊	Yes	45	86.5											1	33.3	3	75			3	100	7	70	1	100	5	71.4	7	87.5	8	100	21	87.5	1	100	6	100	4	80	6	100	17	94.4	0.193
FU024	District supervisor used newborn and stillbirth data quality checklist ◊	Yes	40	76.9											1	33.3	3	75			3	100	7	70	1	100	4	57.1	7	87.5	7	87.5	19	79.2	1	100	5	83.3	3	60	5	83.3	14	77.8	0.559
FU025	District supervisor discussed health facility's performance ◊	Yes	41	78.8											2	66.7	3	75			2	66.7	7	70	1	100	3	42.9	6	75	8	100	18	75	1	100	6	100	4	80	5	83.3	16	88.9	0.445
FU026	District supervisor discussed actions with respondents ◊	Yes	38	73.1											1	33.3	3	75			2	66.7	6	60	1	100	3	42.9	5	62.5	7	87.5	16	66.7	1	100	6	100	4	80	5	83.3	16	88.9	0.379
FU027	District supervisor sent supervisory visit report ◊	Yes	29	55.8											2	66.7	1	25			1	33.3	4	40	1	100	3	42.9	7	87.5	6	75	17	70.8	0	0	2	33.3	3	60	3	50	8	44.4	0.124
	Average PRISM score on quality of supervision (%) □		74.23										0		46.67		65.00				73.33		62.00		100.00		51.43		80.00		90.00		75.83		80.00		83.33		72.00		80.00		78.89		

Notes: * Supportive supervision at data office, data from Tool 4; ‡ Supportive supervision at health facility, data from Tool2b; ◊ indicators collected for health facilities with 1 or more supervisory visits in the last 3 months and contributing to the PRISM score on quality of supervision; □ score calculated for health facilities with 1 or more supervisory visits in the last 3 months according to the PRISM user's kit, formulas are reported in Appendix S6; § comparison of frequencies across countries.

Abbreviations: CAR = Central African Republic; EN-MINI Tools= Every Newborn-Measurement Improvement for Newborn & Stillbirth Measures Tools; PRISM = Performance of Routine Information System Management; RHIS = Routine Health Information System

Appendix S11. End-users' perspectives

A) Figure: End-users' perspectives

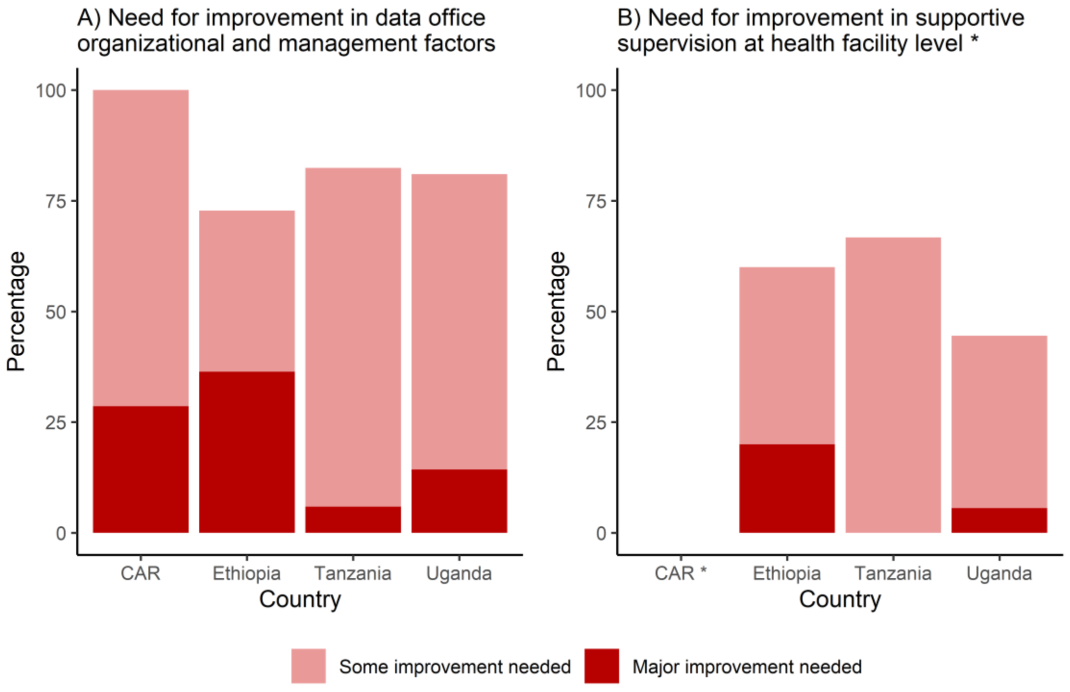

Notes: *measures collected for health facilities with one or more supervisory visits in the last 3 months, CAR frequencies not available due to supervision not performed (see **Figure 3, Appendix S10** in the Online Supplementary Document).
Abbreviations: CAR = Central African Republic

B) Table: End-users' perspectives

Variable identifier in EN-MINI tools		Overall		CAR: Bangui City Administration		CAR: Health region 1		CAR: Health region 2		CAR: Health region 7		Total CAR	
		n	%	n	%	n	%	n	%	n	%	n	%
MAT112.1.1	**Need for improvement in organizational and management factors ***	N=56		N=1		N=1		N=2		N=3		N=7	
	Any improvement needed (some or major improvement)	46	82.1	1	100	1	100	2	100	3	100	7	100
	No improvement needed	10	17.9	0	0	0	0	0	0	0	0	0	0
	Some improvement needed	36	64.3	1	100	1	100	2	100	1	33.3	5	71.4
	Major improvement needed	10	17.9	0	0	0	0	0	0	2	66.7	2	28.6
FU027.1	**Need for improvement in supportive supervision ◊**	N=52		N=0		N=0		N=0		N=0		N=0	
	Any improvement needed (some or major improvement)	30	57.7										
	No improvement needed	13	25										
	Some improvement needed	27	51.9										
	Major improvement needed	3	5.8										
	Missing	9	17.3										

Variable identifier in EN-MINI tools		Ethiopia: Addis Ababa City Administration		Ethiopia: Oromia		Ethiopia: Amhara and Gambella		Ethiopia: South Ethiopia and Sidama		Total Ethiopia	
		n	%	n	%	n	%	n	%	n	%
MAT112.1.1	**Need for improvement in organizational and management factors ***	N=2		N=3		N=2		N=4		N=11	
	Any improvement needed (some or major improvement)	1	50	2	66.7	1	50	4	100	8	72.7
	No improvement needed	1	50	1	33.3	1	50	0	0	3	27.3
	Some improvement needed	1	50	0	0	0	0	3	75	4	36.4
	Major improvement needed	0	0	2	66.7	1	50	1	25	4	36.4
FU027.1	**Need for improvement in supportive supervision ◊**	N=3		N=4		N=0		N=3		N=10	
	Any improvement needed (some or major improvement)	0	0	3	75			3	100	6	60
	No improvement needed	0	0	1	25			0	0	1	10
	Some improvement needed	0	0	2	50			2	66.7	4	40
	Major improvement needed	0	0	1	25			1	33.3	2	20
	Missing	3	100	0	0			0	0	3	30

Variable identifier in EN-MINI tools		Tanzania: Dar es Salaam City Administration		Tanzania: Iringa N=6		Tanzania: Shinyanga N=4		Tanzania: Simiyu N=6		Total Tanzania	
		n	%	n	%	n	%	n	%	n	%
MAT112.1.1	**Need for improvement in organizational and management factors ***	N=1		N=6		N=4		N=6		N=17	
	Any improvement needed (some or major improvement)	0	0	6	100	3	75	5	83.3	14	82.4
	No improvement needed	1	100	0	0	1	25	1	16.7	3	17.6
	Some improvement needed	0	0	6	100	3	75	4	66.7	13	76.5
	Major improvement needed	0	0	0	0	0	0	1	16.7	1	5.9
FU027.1	**Need for improvement in supportive supervision ◊**	N=1		N=7		N=8		N=8		N=24	
	Any improvement needed (some or major improvement)	1	100	5	71.4	4	50	6	75	16	66.7
	No improvement needed	0	0	2	28.6	4	50	2	25	8	33.3
	Some improvement needed	1	100	5	71.4	4	50	6	75	16	66.7
	Major improvement needed	0	0	0	0	0	0	0	0	0	0
	Missing	0	0	0	0	0	0	0	0	0	0

Variable identifier in EN-MINI tools		Uganda: Kampala City Administration		Uganda: Lango		Uganda: Karamoja		Uganda: West-Nile		Total Uganda		p-value §
		n	%	n	%	n	%	n	%	n	%	
MAT112.1.1	**Need for improvement in organizational and management factors ***	N=1		N=6		N=8		N=6		N=21		
	Any improvement needed (some or major improvement)	1	100	4	66.7	6	75	6	100	17	81	0.629
	No improvement needed	0	0	2	33.3	2	25	0	0	4	19	0.629
	Some improvement needed	1	100	4	66.7	3	37.5	6	100	14	66.7	0.209
	Major improvement needed	0	0	0	0	3	37.5	0	0	3	14.3	0.157
FU027.1	**Need for improvement in supportive supervision ◊**	N=1		N=6		N=5		N=6		N=18		
	Any improvement needed (some or major improvement)	0	0	0	0	4	80	4	66.7	8	44.4	0.736
	No improvement needed	1	100	0	0	1	20	2	33.3	4	22.2	0.736
	Some improvement needed	0	0	0	0	3	60	4	66.7	7	38.9	0.832
	Major improvement needed	0	0	0	0	1	20	0	0	1	5.6	0.041
	Missing	0	0	6	100	0	0	0	0	6	33.3	0.003

Notes: * data from Tool 4; ◊ indicators collected for health facilities with one or more supervisory visits in the last 3 months (see Appendix S10); § comparison of frequencies across countries

Abbreviations: CAR = Central African Republic; EN-MINI Tools= Every Newborn-Measurement Improvement for Newborn & Stillbirth Measures Tools
